# Simultaneous femoral and tibial lengthening for severe limb length discrepancy in fibular hemimelia

**DOI:** 10.1186/s13018-023-04229-y

**Published:** 2023-11-08

**Authors:** Hosam Mohamed Ghaly, Mahmoud Abdel-Monem El-Rosasy, Abdelhakim Ezzat Marei, Osama Ali El-Gebaly

**Affiliations:** Orthopedic Department, Tanta University, Tanta, Egypt

**Keywords:** Fibular Hemimelia, Limb lengthening, Ilizarov

## Abstract

**Background:**

Fibular Hemimelia (FH) is the most common longitudinal limb deficiency. Significant limb length discrepancy (LLD) will necessitate long treatment times and multiple settings to compensate for LLD when associated with femoral shortening. This study evaluates the outcome of simultaneous femoral and tibial lengthening using the Ilizarov frame.

**Methods:**

This retrospective study included the cases of 12 children with severe limb length discrepancy caused by combined FH and ipsilateral femoral shortening from May 2015 to August 2022. The total LLD ranged from 7 to 14.5 cm. All patients underwent single-session femoral and tibial lengthening using the Ilizarov ring external fixator technique. Additional procedures were performed in the same setting, including Achilles tendon lengthening, fibular anlage excision, peroneal tendons lengthening, and iliotibial band release. Follow-up ranged from 2 to 4 years.

**Results:**

The planned limb lengthening was achieved in ten cases (83%). No cases of joint subluxation or dislocation were encountered. No neurovascular injury has occurred during the treatment course. In all cases, the bone healing index was better on the femoral side than on the tibia. Poor regeneration and deformity of the tibia occurred in two cases (16.6%).

**Conclusion:**

Simultaneous femoral and tibial lengthening using the Ilizarov fixator is a relatively safe procedure with the result of correction of total LLD in one session in a shorter time and less morbidity.

## Introduction

Fibular Hemimelia (FH) refers to partial or complete deficiency of the fibula. It is considered the commonest longitudinal deficiency of long bones, with an incidence ranging from 8 to 20 per million live births [1].

FH is frequently associated with a wide range of anomalies and deformities, e.g., femoral shortening, knee valgus, anteromedial tibial bowing, knee and ankle instability, equino-valgus foot deformities and absence of the lateral rays of the foot [2, 3]. Combining fibular deficiency with ipsilateral femoral shortening may result in severe limb length discrepancy [4].

Isolated lengthening of the tibia carries a risk of knee dislocation. The knee is inherently unstable due to congenital deficiency of cruciate ligaments and bone hypoplasia [5, 6]. Moreover, tibial lengthening may be insufficient to correct a significant LLD in one setting and consequently may necessitate multiple operations to achieve limb equalization. This may be associated with psychological and economic burdens and numerous complications [7].

The objective of this study is to evaluate the rationale of treatment of FH patients suffering from severe limb length discrepancy by simultaneous femoral and tibial lengthening using the Ilizarov frame in terms of safety and efficacy to equalize limb length.

## Materials and methods

This retrospective study included the cases of twelve children with unilateral FH who had severe unilateral lower limb shortening. FH was diagnosed clinically and by imaging studies. The anomaly was associated with ipsilateral femoral shortening of variable severity. Severe limb length discrepancy (LLD) was diagnosed when limb shortening exceeded six cm [8–10]. All patients were treated at the Unit of Limb Reconstruction and Pediatric Orthopaedics from May 2015 to August 2022. The results were reported after a minimum of two years of follow-up.

### Inclusion criteria

Cases with fibular hemimelia aged ≥ 3 years old with a total LLD ≥ 6 cm.

### Exclusion criteria

Excluded from the study were cases of FH associated with congenital femoral deficiency, where the projected LLD at skeletal maturity was over 25 cm (calculated by the multiplier method) [11]. Cases of unstable hips (center–edge angle below 25 degrees) who did not have a hip stabilizing procedure were also excluded from the study.

The demographic data of the study cases are presented in Table 1.

**Table 1:** The demographic data of the study cases

	Age (y)	Gender	Type of FH	Side	Number of foot rays	Total shortening (cm)	Femoral shortening (cm)	Tibial shortening (cm)	LLD at maturity
1	7	F	IB	Rt	4	12	3	9	17.2
2	9	F	IB	Rt	4	13	4	9	17.9
3	5	M	II	Rt	3	7	4	3	12.7
4	11	M	II	Rt	3	14	5	9	17.4
5	3	M	II	Lt	5	6	2	4	13.4
6	4	M	II	Lt	4	9	3	6	18
7	3	F	II	Rt	3	7.5	3	4.5	14.9
8	5	M	IB	Lt	5	9	4	5	16.4
9	7	M	II	Lt	3	10	4	6	15.7
10	4	F	IB	Lt	5	8	3	5	14.6
11	12	M	II	Rt	2	14.5	6	18.5	16.4
12	7	M	II	Rt	4	12	4	8	18.8

Y —years, F— Female, M —Male, FH —fibular hemimelia, Rt —right, Lt —left, cm —centimeter, LLD —Limb length discrepancy

### Preoperative evaluation

General examination was performed to exclude other associated congenital anomalies. Neuromuscular assessment and examination of the hip, knee, and ankle (to detect associated joint instability) were done.

**Fig. 1:**
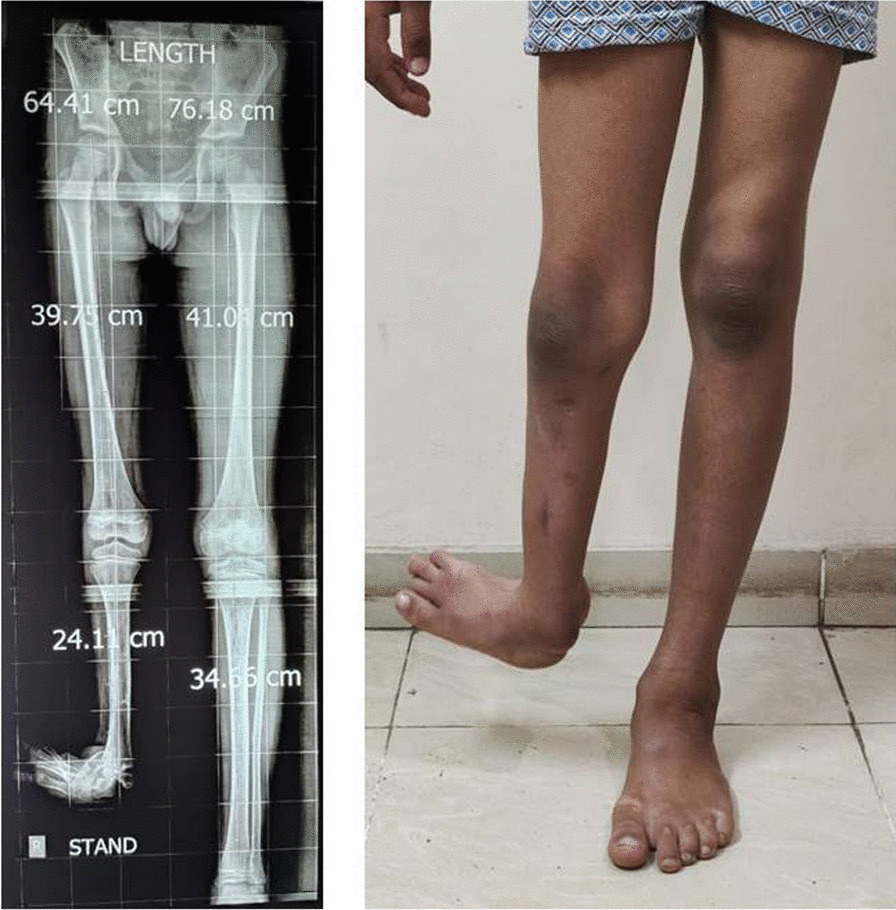
A full-length standing X-ray scanogram was obtained in all cases, with blocks under the short limb to equalize the length

A full-length standing X-ray scanogram was obtained in all cases, with blocks under the short limb to equalize the length. That was done to measure the whole limb length and isolated femoral and tibial lengths (Fig. 1). Magnetic resonance imaging (MRI) was not needed even if there was preoperative knee instability, as we protect the knee joint during lengthening by connecting the tibial and femoral frames. MRI could be done only if there was preoperative patellar instability or if a planned knee reconstructive surgery was indicated, which was beyond the scope of this research.

### Surgical technique

The procedure was performed under general anesthesia with the patient in the supine position. The whole lower limb and ipsilateral hemipelvis were prepped and draped. A sterile tourniquet was applied to the upper thigh. Percutaneous fibular osteotomy was done using a drill bit and an osteotome. If the fibula was absent, a segment of the fibrous fibular anlage was excised.

**Fig. 2:**
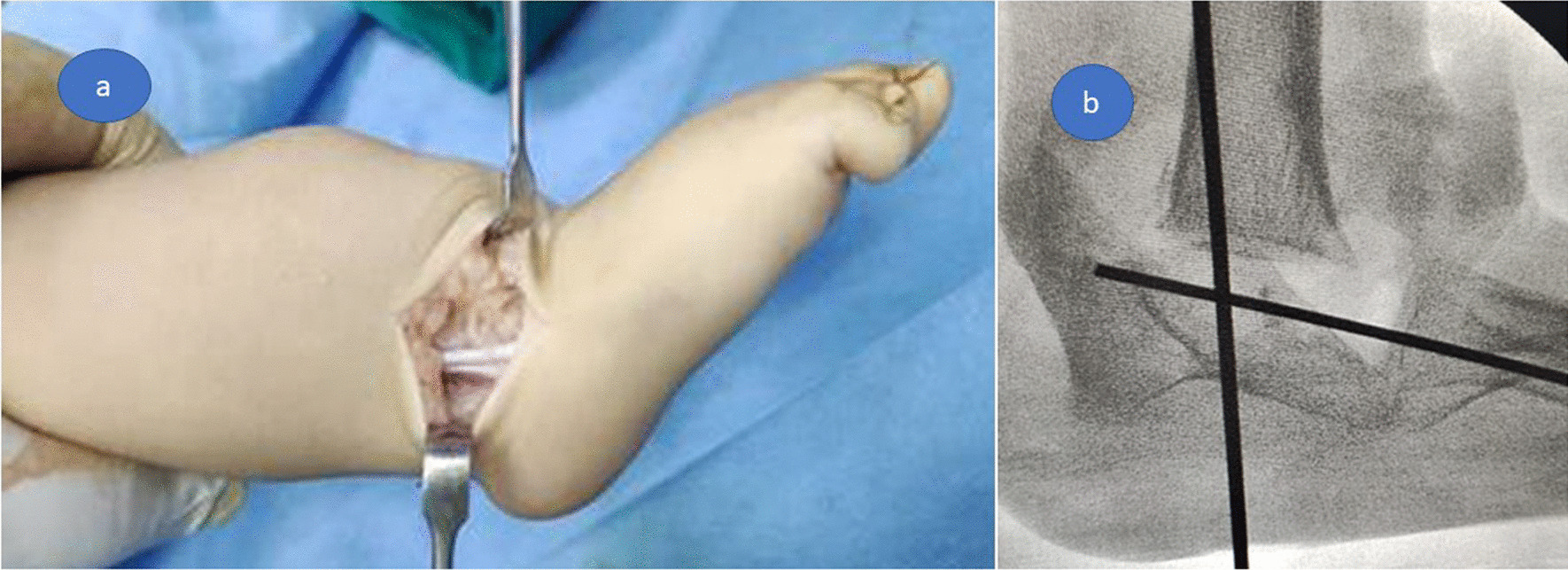
**a** a Z-lengthening of Achilles and peroneal tendons through lateral approach, **b** fixation by a trans-calcaneal Kirschner wire

A Z-lengthening was done to the Achilles and peroneal tendons through a lateral approach to the ankle and hindfoot (Fig. 2a). An osteotomy to separate the fused talus and calcaneus was performed, and then the foot was repositioned plantigrade under the tibia and fixed by a trans-calcaneal Kirschner wire (Fig. 2b). The tourniquet was released for hemostasis, and the wound was closed in layers.

In cases presented with ankle dislocation, as a consequence of a previous lengthening procedure, the foot was centralized under the tibia by doing a tibiotalar fusion after resection of the lower end tibia to allow a safe acute deformity correction.

A pre-assembled two-ring Ilizarov frame was applied to the femur. Percutaneous release of the Iliotibial band was done, and from the same incision, a distal metaphyseal femoral osteotomy was done. The valgus deformity of the distal femur was then corrected acutely.

**Fig. 3:**
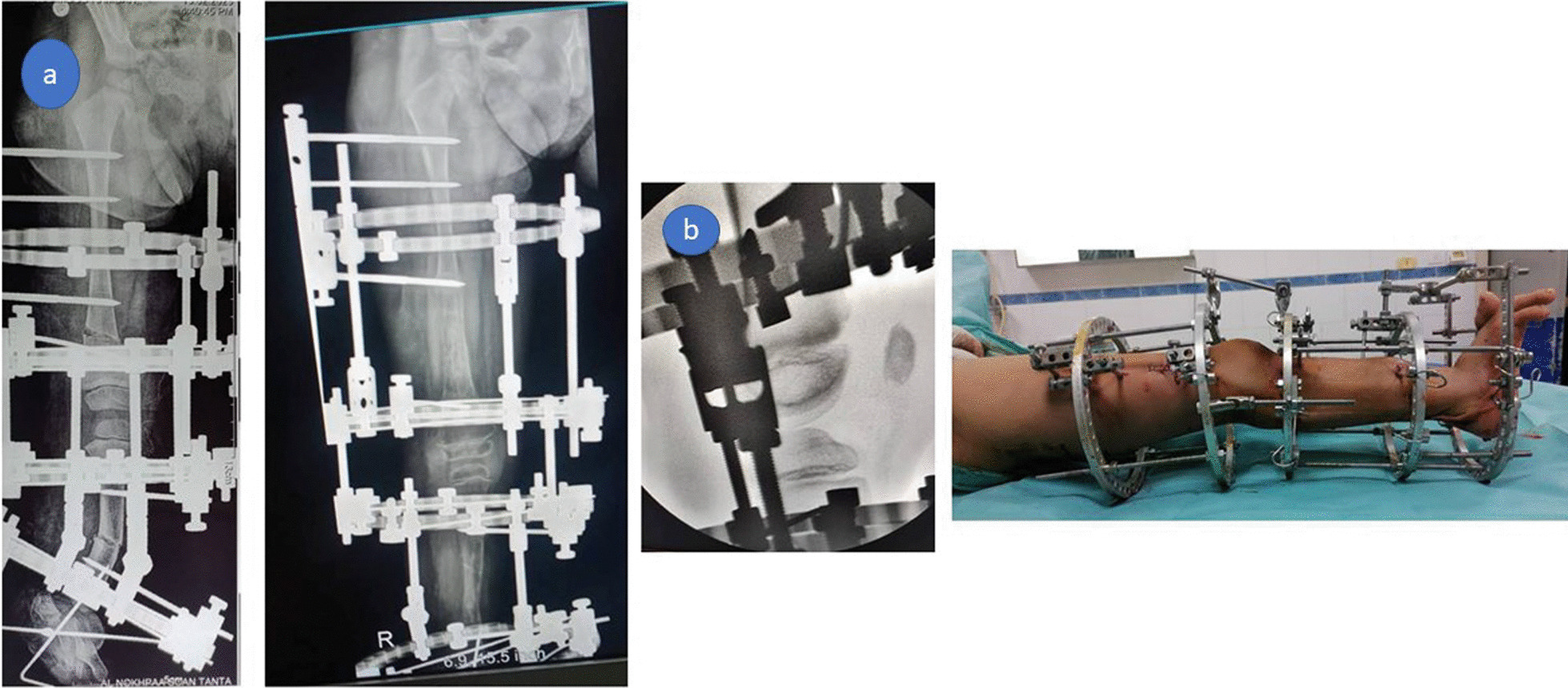
**a** Tibial and femoral frames were applied, distal femoral osteotomy for lengthening. Tibial osteotomy was done at the level of CORA for simultaneous correction and lengthening, **b** The femoral and tibial frames were connected using universal hinges with the knee fully extended

Another Ilizarov frame was then applied to the leg with the foot included in the frame. The foot was fixed plantigrade relative to the distal tibia. A percutaneous tibial osteotomy was performed at the level of tibial angulation (Fig. 3a).

The femoral and tibial frames were connected using universal hinges with the knee fully extended to avoid knee subluxation. Image intensifier control was mandatory to monitor deformity correction. The hinges were locked by a threaded rod fixed anteriorly to the rings closest to the knee (Fig. 3b).

### Postoperative management

Distraction of the lengthening osteotomies was started on the fifth postoperative day at a rate of one mm per day for each femoral and tibial osteotomies. After one week of distraction, plain X-rays were obtained to ensure proper distraction of the osteotomies. The femoral osteotomy distraction was continued at the same rate of one mm. per day, while the tibial osteotomy distraction was slowed down to half mm per day.

All patients were followed in the outpatient clinic every two weeks during the distraction phase, and new X-rays were obtained at each visit. They were evaluated clinically for treatment-related problems like pin tract infection and patients’ tolerance to the procedure. The X-rays were examined for the quality of regenerate bone formation, progress of deformity correction, signs of pin loosening, and early signs of hip or knee subluxation.

During the consolidation phase, the outpatient clinic visits were reduced to every month until radiological consolidation of the lengthening regeneration. The fixator was removed under general anesthesia with great care to avoid breakage of the newly formed bone. A well-molded unilateral hip spica cast was applied and kept for 4–6 weeks. The child was allowed to bear weight as tolerated in the spica cast. After cast removal, a hinged hip-knee-ankle foot orthosis was applied, and the child was encouraged to walk and actively move his knee and ankle. Guided physiotherapy and rehabilitation were then gently started to regain the joints’ range of motion and muscle strength.

The patients were followed every three months for one year, then every six months.

The final follow-up period ranged from 24 to 50 months.

### Evaluation of the outcome

**Fig. 4:**
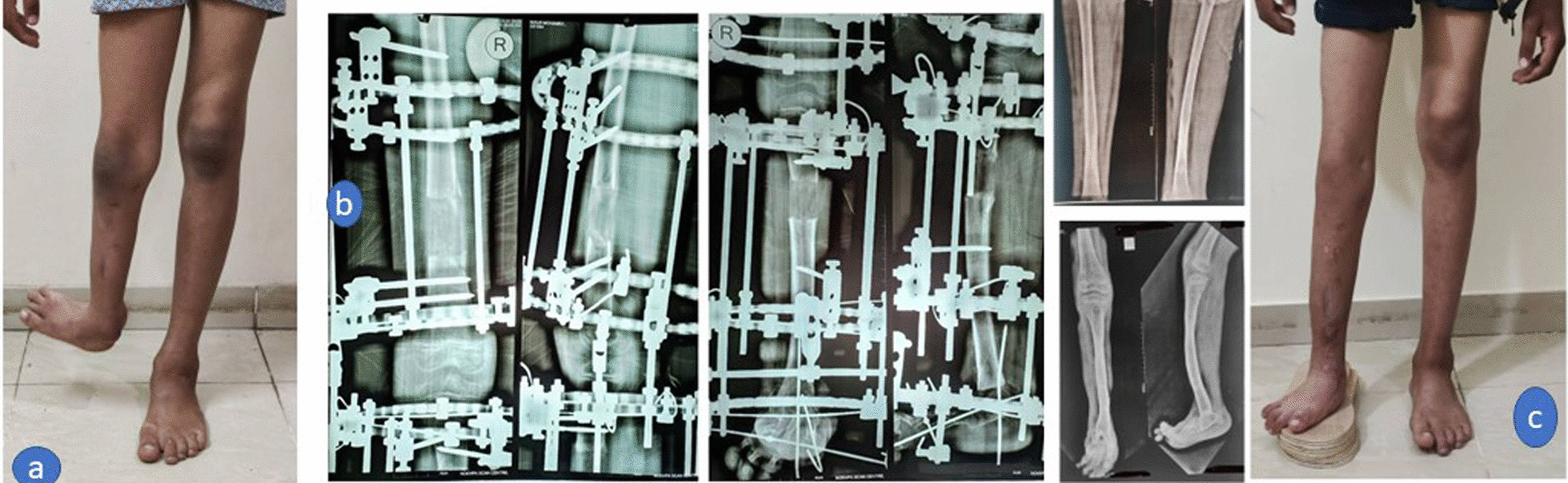
**a** Preoperative clinical image with LLD 8 cm, **b** combined femoral and tibial lengthening with better regenerate in the femur than in the tibia, **c**) Final correction of the foot and limb length discrepancy to less than 2.5 cm (satisfactory outcome)

Criteria of a satisfactory result were set forth as follows: correction of LLD to less than 2.5 cm, (Fig. 4 a, b, c) no residual deformity, maintenance of joint range of motion, and absence of treatment-related problems (permanent joint stiffness, and joint subluxation/dislocation).

**Fig. 5:**
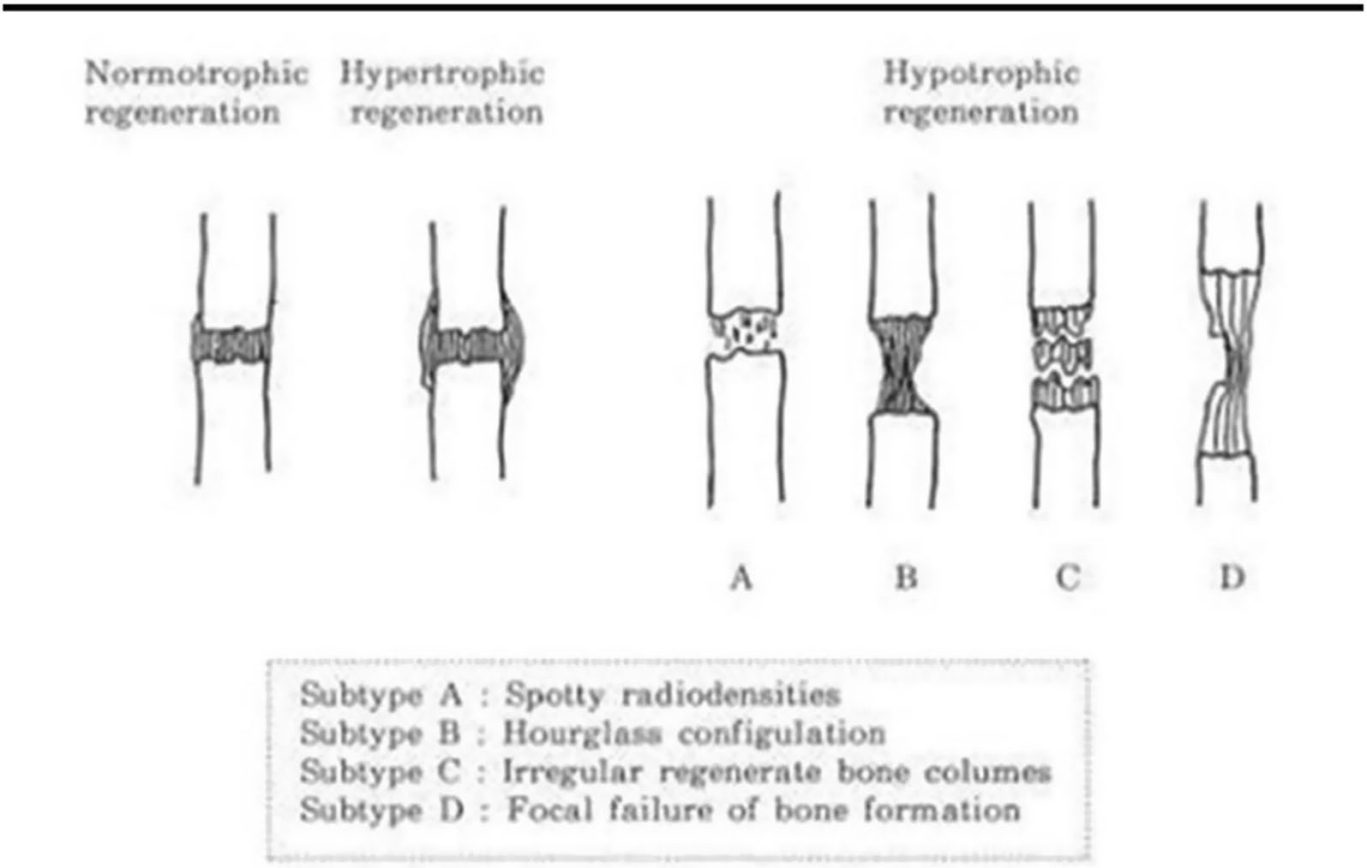
Radiographic classification of the formed bony regenerate (Normotrophic, Hypertrophic and hypotrophic)

The lengthening regenerate was classified radiologically into one of three types (type 1. Hypertrophic regenerate, type 2. Normotrophic regenerate, and type 3. Hypertrophic regenerate) (Fig. 5) [12].

Healing indices were also determined and included:

- External fixation time (EFT): the time passing between insertion and removal of the external fixator.
- External fixation index (EFI): the number of days the external fixator is attached to bone per centimeter of length gained.
- Total treatment time (TTT): the time passing between insertion of the external fixator and removal of the spica cast.
- Bone healing index (BHI): relates the total treatment time to the measured length of bone gained.

Treatment-related difficulties were classified into problems, obstacles, and complications (according to Paley) [13]. Where problems are adverse effects that occur during treatment and are resolved without surgical intervention. Obstacles are adverse effects that occur during therapy but resolve with surgical procedures. Complications are incidents that don’t resolve even with surgical intervention and affect the final treatment results.

## Results

Twelve patients (8 males [66.5%] and 4 females [33.5%]) with FH and ipsilateral femoral shortening constitute the material of our study. All cases underwent simultaneous femoral and tibial lengthening by the Ilizarov frame. The average age at the treatment time was 6.75 years (median: 6), (range: 3–12 years). The left lower limb was short in five cases (42%), while the right limb was affected in seven cases (58%). Eight patients (66.5%) had type II FH, and four patients (33.5%) had type IB according to Kalamchi–Achterman classification of FH (**Type IA**: the proximal fibular physis distal to the proximal tibial one and distal fibular physis is proximal to the talar dome, **Type IB**, more severe fibular deficiency and no distal support of the ankle joint, **Type II**, complete fibular absence. [4].

The total LLD ranged from 6 to 14.5 cm (average 10.2 cm, median: 9.5). The average femoral shortening was 3.7 cm (range 3–6 cm, median 4 cm), while the average tibial shortening was 6.5 cm (range 4–9 cm, median: 6 cm).

The average follow-up period after bone healing was 37 months (range 24 to 50 months).

The total limb lengthening achieved with our treatment averaged 9.8 cm (range 6–13 cm). Length gained from the femur ranged from 3 to 8 cm (average 5.9 cm, median 5.5 cm), and from the tibia ranged from 3 to 5 cm (average 3.9 cm, median 4 cm).

The healing indices are detailed in Table 2

**Table 2:** Healing indices of the study cases

Case no.	EFT (m)	EFI (m/cm)		TTT (m)	BHI (m/cm)	
		Fem	Tib		Fem	Tib
1	8	1.1	1.6	10	1.4	2
2	10	1.3	2	12	1.5	2.4
3	5	1.2	1.6	6.5	1.6	2.7
4	9	1.1	2.3	12	1.5	3
5	4	1.3	1.3	5.5	1.8	1.8
6	6	1.2	1.5	8	1.6	2
7	6	1.5	2	7.5	1.9	2.5
8	6	1	2	8	1.3	2.6
9	7	1.2	1.8	9	1.5	2.3
10	6	1.2	2	8	1.6	2.6
11	10	1.3	2.2	12	1.5	2.7
12	8.5	1.2	1.7	11	1.6	2.2
A	7.1	1.25	1.8	8.75	1.6	2.4
M	7.5	1.2	1.8	7.75	1.55	2.4

EFT —external fixator time, m = months, EFI —external fixation index, m/cm = months per centimeter, TTT —total treatment time. BHI —bone healing index, F —femur, Tib —tibia, A —average, M —median

The femoral regeneration was hypertrophic in two cases (16.7%) and Normotrophic in the remaining ten cases (83.3%). Normotrophic tibial regeneration was detected in only three patients (25%) and Hypotrophic in the remaining nine cases (75%).

The planned limb lengthening was achieved in ten cases (83%). Residual limb shortening of more than 2.5 cm was accepted in two patients (17%) due to poor and hypotrophic tibial regeneration. In these two cases, we had to slow down tibial lengthening and do compression-distraction of the regenerate “accordion mechanism” to enhance bone consolidation. Bone grafting was not needed in any case.

Treatment-related difficulties are summarized in Table 3

**Table 3:** Treatment-related difficulties

Case no.	Pin tract infection	Insufficiency fracture	Knee stiffness	Knee dislocation	Superficial skin necrosis	Neurovascular injuries	Valgus deformity of the regenerate	Significant LLD
1	*		*					
2	*		*					
3	*		*					
4	*		*				*	*
5	*		*					
6	*	*	*					
7	*		*					
8	*		*					
9	*		*					
10	*		*					
11	*		*		*		*	*
12	*		*					

LLD — limb length discrepancy

**Fig. 6:**
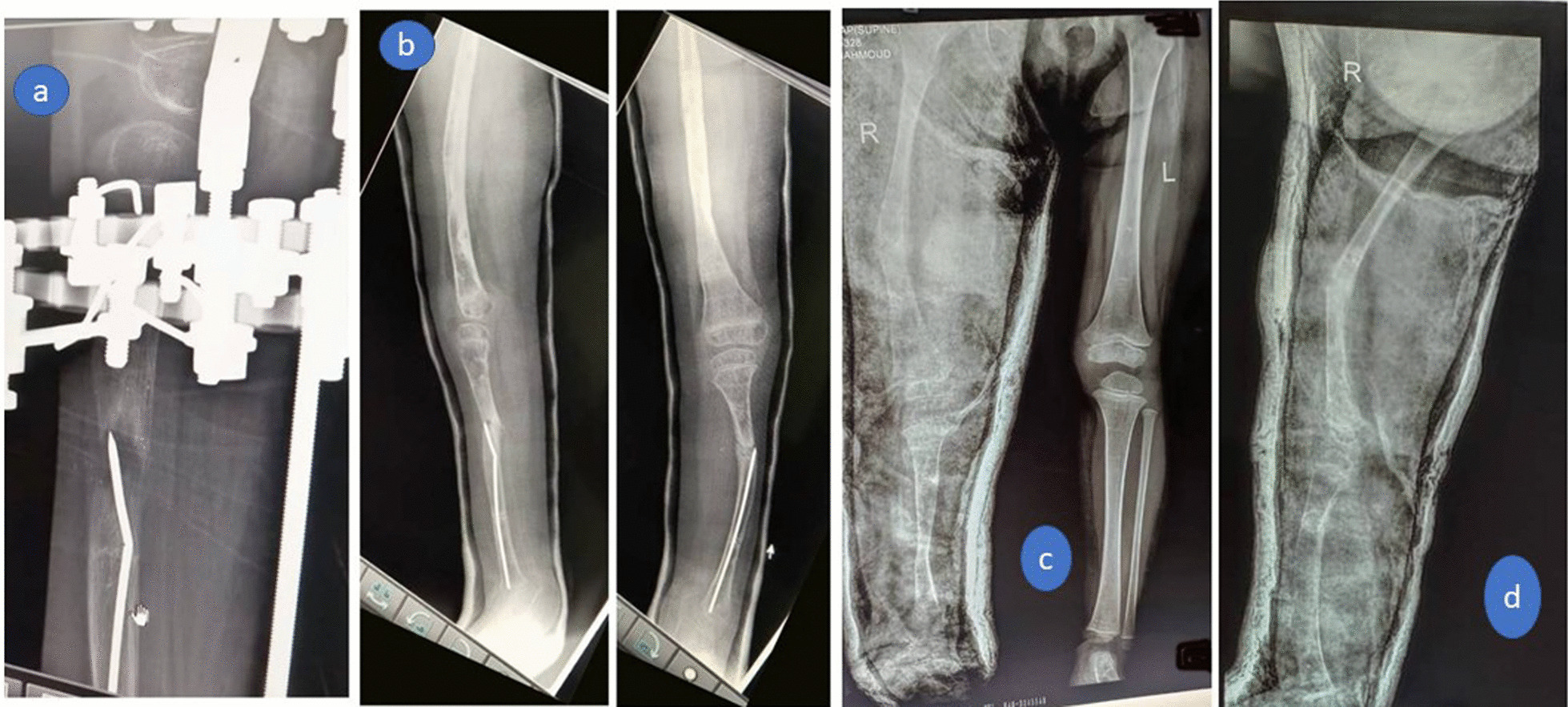
**a** Valgus deformity occurred at the tibial regenerate, which was managed by **b** corrective osteotomy and intramedullary rodding, **c** anteroposterior, and d) lateral x-ray images of Insufficiency fracture of the femur at the site of a previous pin

Pin tract infection occurred in all cases and was resolved with pin-site care and antibiotic treatment. No case required debridement. Knee stiffness occurred in all cases, intensive physiotherapy was performed after brace removal and full range of knee motion was regained at the time of final follow-up. No residual joint stiffness, subluxation, or dislocation occurred in any case. Superficial skin necrosis occurred in only one case (8%) at the site of the foot skin incision and was resolved by daily dressing. In two (17%) cases, valgus deformity occurred at the tibial regenerate, managed by corrective osteotomy and intramedullary rodding (Fig. 6a,b). This resulted in residual LLD of 2 cm, which could be tolerated (non-significant). Insufficiency femoral fracture at a site of previous pin happened in one case which could be treated conservatively by applying above knee cast for 1 month. (Fig. 6c, d) No complications were encountered, e.g., neurovascular injuries, compartment syndrome, knee dislocation, significant residual LLD, or regenerate fracture.

At the final follow-up, the outcome was satisfactory (according to our criteria) in ten cases 83% and unsatisfactory (due to residual LLD of more than 2.5 cm) in two cases 17%.

A sensible statistical analysis was impossible because of the small number of cases.

## Discussion

Combining FH and congenital short femur may cause significant lower limb shortening. An LLD of six cm or more is considered severe according to the criteria of McCaw and Bates [8]. Isolated tibial lengthening for such LLD is challenging because of the treatment-related difficulties, regenerate failure, pin tract infection, muscle contractures, joint stiffness, and the psychological burden for patients and their caregivers. That is why some authors recommended early amputation and prosthetic fitting for cases of LLD more than 7.5 cm, having the advantages of a single surgery, early ambulation, short hospital stay, limb length equalization, and quick return to activities [13, 14]. However, amputation is a “no way back” procedure and may result in major psychological drawbacks. It also causes a lack of proprioception sensation and requires periodic prosthesis changes.

In most eastern countries, amputation is not an acceptable procedure by the public “considered as a social stigma,” and the parents would accept a short limb rather than having amputation performed for their kids.

The current study evaluated the results of using the Ilizarov external fixator to correct severe LLD as an alternative to amputation. Simultaneous femoral and tibial lengthening using the Ilizarov frame was performed to shorten the treatment period, divide the whole lengthening into two bony segments rather than one, lessen the number of operations, achieve a more significant gain in length, and decrease treatment-related complications.

The range of follow-up period was two to four years, which was enough to evaluate the lengthening procedure only in managing FH as we did not assess the whole management of FH. The youngest patient was three years old and required follow-up for 2 to 4 years, and after that, the patient would require another lengthening procedure, which was beyond the scope of this study.

In our present study, the new regenerate formation and consolidation were weaker and slower at the tibial level, i.e., Hypotrophic. On the other hand, the femoral lengthening regeneration was well formed and rapidly consolidated, i.e., Normotrophic or even hypertrophic. This could be attributed to the better blood supply and soft-tissue (muscular) envelope around the femur than the tibia, a subcutaneous bone with precarious blood supply. This vital note directed us to keep the distraction of tibial osteotomy at a slower rate than usual (0.5 mm per day divided into 2 sessions). Meanwhile, the femoral distraction rate was 1 mm per day.

Moreover, this led us to the rationale that the femur can compensate for a larger amount (longer segment) of LLD rather than the tibia, despite that most of the shortening distance was on the tibial level in all cases. The final result was the equalization of LLD but with different knee levels that were non-significant for the patients and their parents. This concept of over-lengthening of the femur and under-lengthening of the tibia to equalize the net LLD was better than going through another session of tibial lengthening with more complications and psychological burden to the patient.

Femoral new regenerate was classified as normotrophic in ten cases and hypertrophic in only two, according to Vade’s classification [12]. On the other hand, the tibial new regenerate was classified as hypotrophic in nine cases and normotrophic in three cases. For the hypotrophic regenerate, we had to slow the rate of tibial distraction to be “day after day” instead of daily distraction. We also used the “accordion technique” [14] to improve the quality and consolidation of the poor newly-formed tibial regenerate. However, no bone grafting was needed in any case.

EFI and BHI for the femur were less (better) than those for the tibia in all cases. This result corresponds to our concept that the femur is much better in regenerate formation than the tibia. We, therefore, applied our rationale of gaining most of the LLD correction through the femur rather than the tibia. This differs from Bishay’s concept [15], which lengthened the bony segment by the same amount of shortening regardless of the contribution to the whole LLD.

Among all the adverse events during the follow-up period, only two obstacles needed another session to manage. They were in the form of valgus deformation of the tibia in two patients at the regenerate site after splint removal due to weak regeneration. Both patients underwent corrective osteotomy and intramedullary rod application to maintain correction until complete consolidation. The other adverse events were classified as problems that needed no operative interference. Problems included pin tract infections that responded to pin site care and systemic antibiotics. All patients experienced stiffness of the knee, which improved with intensive physiotherapy.

Barker et al. [5] published their study on 35 patients (23 males and 12 females) with a mean age of 22 years. Their cases underwent femoral lengthening by the Ilizarov method. The observed recovery pattern of knee ROM showed that 88% of knee flexion was regained by 6 months, 92% by 12 months, and 97% by 18 months. Of the 35 patients in their study, all but three cases regained or improved their preoperative ROM. None of the patients lost more than 10 degrees of flexion. Their statistical analysis showed that lengthening one bone or more than one bone simultaneously had no impact on the loss of knee flexion range.

Bowen et al. [16] carried out simultaneous ipsilateral femoral and tibial lengthening using the Wagner technique, and they reported a high rate of knee subluxation (3 out of 10 cases). Primarily due to a lack of monolateral fixators connection to protect the unstable knees in fibular hemimelia. On the other hand, Curran et al. [17] utilized the Ilizarov device with linked frames throughout the lengthening process, and they reported no cases of subluxation, but two of the patients developed an extension contracture of the knee that required a quadricepsplasty. Choi et al. [18] reported repeated tibial lengthening using Wagner’s technique with good outcomes, probably due to small repeated lengthening “dozed lengthening” to avoid complications of tissue contracture with excessive lengthening.

The current study achieved single-session femoral and tibial lengthening with similar outcomes. Miller et al. [19] reported satisfactory results in 12 fibular deficiencies with the Ilizarov technique. McCarthy et al. [20] achieved limb equalization and gait with minimal complications through the Ilizarov technique, which is comparable to our current study.

Limitations of our study include its retrospective nature, a small number of the cases, which is attributed to the rare nature of these cases (Fibular hemimelia combined with congenital femoral deficiency and associated with severe limb length discrepancy).

## Conclusion

In light of our treatment results and literature review, severe LLD in cases of FH with combined tibial and femoral shortening can be managed by simultaneous femoral and tibial lengthening using Ilizarov external fixator techniques. The advantages of performing simultaneous lengthening include reduced morbidity and psychological trauma, reduced number of operations and economic cost, less total external fixation time and bone healing index by dividing the entire lengthening goal between the femur and tibia, and more rapid recovery when compared to performing the operations sequentially. Complication rate and severity were not higher than sequential lengthening. However, the patients and/or the parents should have sufficient knowledge about the potential problems, lengthy treatment, and the possibility of a second surgery.

## Data Availability

Not applicable.
